# High salt diet accelerates skin aging in wistar rats: an 8-week investigation of cell cycle inhibitors, SASP markers, and oxidative stress

**DOI:** 10.3389/fbioe.2024.1450626

**Published:** 2024-10-11

**Authors:** Xile Peng, Nannan Liu, Baihan Zeng, Yilin Bai, Yang Xu, Yixiao Chen, Li Chen, Lina Xia

**Affiliations:** ^1^ School of Health Preservation and Rehabilitation, Chengdu University of Traditional Chinese Medicine, Chengdu, China; ^2^ State Administration of Traditional Chinese Medicine Key Laboratory of Traditional Chinese Medicine Regimen and Health, Chengdu University of Traditional Chinese Medicine, Chengdu, China; ^3^ Key Laboratory of Traditional Chinese Medicine Regimen and Health of Sichuan Province, Chengdu, China

**Keywords:** high salt diet, skin aging, oxidative stress, SASP, cell cycle inhibitors

## Abstract

**Background:**

Recent studies have shown that the high salt diet (HSD) is linked to increased dermal pro-inflammatory status and reduced extracellular matrix (ECM) expression in inflamed skin of mice. Decreased ECM content is a known aging phenotype of the skin, and alterations in ECM composition and organization significantly contribute to skin aging. This study aimed to determine whether a high salt diet accelerates skin aging and to identify the time point at which this effect becomes apparent.

**Methods:**

Wistar rats were randomly divided into normal diet and high salt diet groups and fed continuously for 8 weeks. Skin samples were collected at weeks 7 and week 8. Skin pathological sections were evaluated and levels of cell cycle inhibitors, senescence-associated secretory phenotype (SASP), oxidative stress and vascular regulatory factors (VRFs) were examined. Correlation analyses were performed to reveal the effect of a high salt diet as an extrinsic factor on skin aging and to analyse the correlation between a high salt diet and intrinsic aging and blood flow status.

**Results:**

At week 8, HSD rats exhibited thickened epidermis, thinned dermis, and atrophied hair follicles. The expression of cell cycle inhibitors and oxidative stress levels were significantly elevated in the skin of HSD rats at both week 7 and week 8. At week 7, some SASPs, including TGF-β and PAI-1, were elevated, but others (IL-1, IL-6, IL-8, NO) were not significantly changed. By week 8, inflammatory molecules (IL-1, IL-6, TGF-β), chemokines (IL-8), proteases (PAI-1), and non-protein molecules (NO) were significantly increased. Notably, despite elevated PAI-1 levels suggesting possible blood hypercoagulation, the ET-1/NO ratio was reduced in the HSD group at week 8.

**Conclusion:**

The data suggest that a high salt diet causes skin aging by week 8. The effect of a high salt diet on skin aging is related to the level of oxidative stress and the expression of cell cycle inhibitors. Additionally, a potential protective mechanism may be at play, as evidenced by the reduced ET-1/NO ratio, which could help counteract the hypercoagulable state and support nutrient delivery to aging skin.

## 1 Introduction

Skin aging is a dynamic and continuous process influenced by both genetic and external factors, primarily manifesting as changes in cell morphology and function. It is generally categorized into intrinsic and extrinsic aging (Farage et al., 2008). Intrinsic aging, also known as natural aging, is a process driven by genetic factors. It is mainly due to the accumulation of reactive oxygen species (ROS), a by-product of cellular metabolism, which in turn can cause damage to key cellular components such as cell membranes, enzymes, and DNA (Poljsak et al., 2012). Extrinsic aging, which is caused by the accumulation of damage from the effects of environmental factors (e.g., ultraviolet radiation, environmental pollution) and lifestyle changes (e.g., sleep deprivation, stress, smoking, malnutrition, and alcoholism) (Rittie and Fisher, 2002; Krutmann et al., 2017). This form of aging is marked by skin roughness, loss of elasticity, deep wrinkles, dilated capillaries, and the formation of pigmented spots (Vierkotter et al., 2010; Huls et al., 2016; Morita, 2007).

Currently, it has been demonstrated that a high salt load affects the production of inflammation-associated cytokines and may potentially lead to the remodeling of the skin’s extracellular matrix (ECM). A decrease in ECM content is one of the phenotypes of skin aging, with structural and functional changes in the ECM observed in both endogenous and exogenous aging skin (Todorova and Mandinova, 2020). Numerous studies have shown alterations in the accumulation of type I and type III collagen, changes in the ratio of type I/III collagen (Lovell et al., 1987), impaired synthesis of ECM molecules, and changes in elastic fiber organization in aging skin (Shin et al., 2019). However, ECM changes, as one of the manifestations of skin aging, are not a definitive marker of aging. Therefore, the relationship between the high salt diet (HSD) and skin aging is unclear. This study aimed to determine whether a high salt diet accelerates skin aging and to identify the time point at which this effect becomes apparent.

**FIGURE 1:**
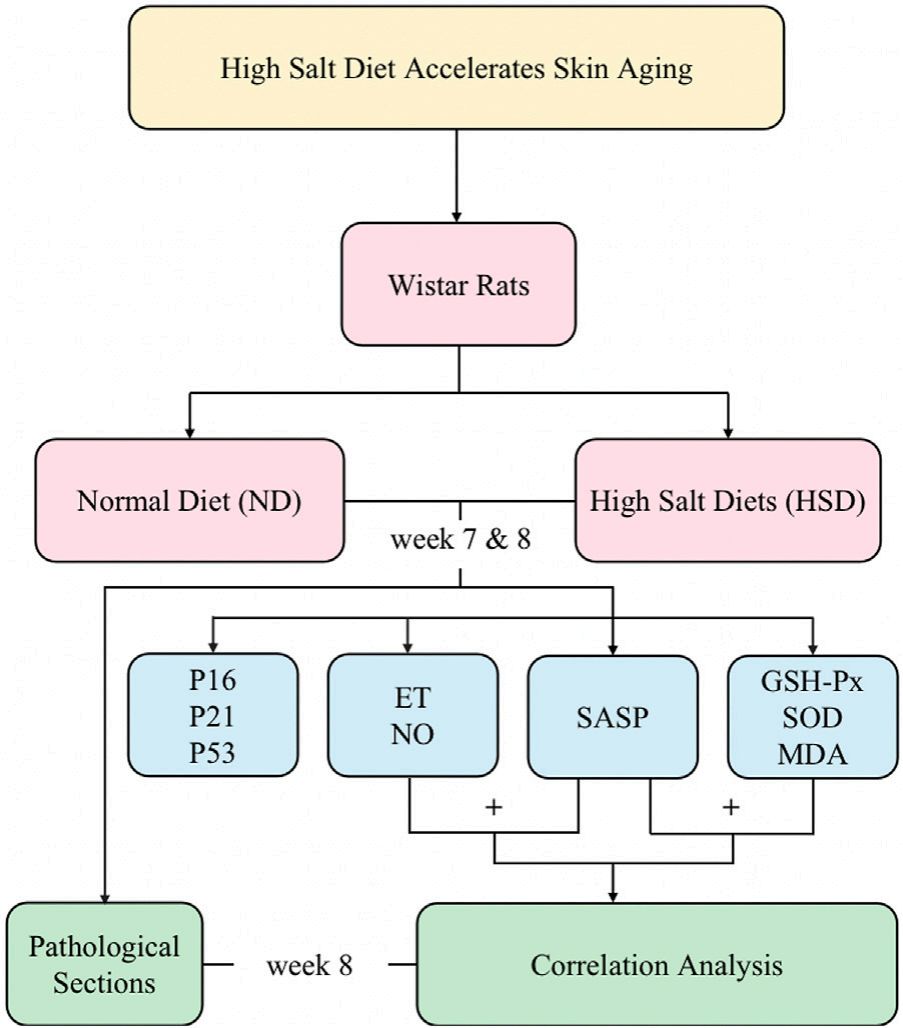
The schematic flowchart of the study.

In this study, we used a high salt diet (8% NaCl) continuously fed to 2-month-old rats for 8 weeks, and the sampling and measurements were performed at week 7 and week 8, respectively. We observed the effect of HSD on the skin senescence phenotype of rats and detected the senescence-associated secretory phenotype (SASP) and cell cycle inhibitors. We clarified that HSD caused skin senescence in rats by week 8. We also examined oxidative stress indicators, and through correlation analyses, determined that skin aging due to high salt is closely related to oxidative stress. Overall, this approach provides a basis for exploring the time points and molecular mechanisms of skin aging due to HSD. The main process of this study is shown schematically in Figure 1.

## 2 Materials and methods

### 2.1 Study animals and diet

24 Wistar rats (200 ± 20 g) were housed in a well-ventilated room at room temperature with a 12-hour light-dark cycle. The rats were purchased from the SiPeiFu (Beijing) Biotechnology Co.,Ltd. (license number: SCXK (Beijing) 2019-0010). After 1 week of acclimatization, the Wistar rats were randomly divided into two groups: normal diet (ND) (0.3% NaCl diet) and high salt diet (HSD) (8% NaCl diet) for 8 weeks. HSD and ND were purchased from Beijing Keao Xieli Feed Co., Ltd. and were numbered as “8% NaCl AIN-76A” and “0.3% NaCl AIN-76A,” respectively. The composition of “8% NaCl AIN-76A” and the proportion of each component are shown in Table 1. The composition of “0.3% NaCl AIN-76A” was the same except for the difference in sodium chloride content.

**TABLE 1:** The composition of 8% NaCl AIN-76A and its proportion.

Formulas	Proportions
Casein	20
Methionine	0.3
Sucrose	50.9
Corn Starch	15
Cellulose	5
Corn Oil	5
Sodium Chloride	8.7
Calcium Hydrogen Phosphate	1.75
Multimineral S10001A	1.75
Choline Bitartrate	0.2
Multivitamin V10001C	0.1
Dye, Blue	0.005
Total	108.705

### 2.2 Sample processing

Rats were anesthetized via intraperitoneal injection of 2% sodium pentobarbital at a dose of 40 mg/kg. Anesthesia was confirmed by the absence of reflex responses. Following confirmation of anesthesia, euthanasia was performed through exsanguination. A disposable blood collection needle (0.55*19 mm) was quickly inserted into the abdominal aorta to collect blood using a vacuum blood collection tube. This procedure ensures that the rat remains in a state of deep anesthesia and is completely pain-free. Subsequently, the vital signs of the animals, including heart rate and respiration, were monitored to confirm death. The blood was allowed to stand at room temperature for 1 h, and after it was allowed to coagulate, it was centrifuged using a high-speed cryogenic centrifuge (TGL-16M) at 3,000 r/min for 15 min, and then the upper layer of serum was aspirated and preserved in 5 mL EP tubes for spare use.

After preparation of the skin, 4 cm^2^ of skin tissue was cut and washed in cold saline and drained with filter paper. 2 cm^2^ of skin tissue was taken and immersed in 4% paraformaldehyde solution for fixation. The remaining samples were homogenised using a multi-tissue tissue grinder (Shanghai Jingxin Industrial Development Co., China). The homogenising medium was PBS (0.01 M, PH 7.4), and the ratio of skin tissue to homogenising medium was 1:9. After homogenisation, the samples were centrifuged at 10,000 × g for 10 min at 4°C, and the supernatant was stored at −80°C.

### 2.3 Skin histopathological examination

The trimmed and flattened tissues were dehydrated and embedded, and paraffin sections of 3 μm thickness were prepared using a slicer (Thermo Scientific). The paraffin sections were successively stained with hematoxylin for 3–5 min and eosin for 5 min, and then dehydrated and blocked (HE staining solution kit purchased from Leagene). The senescence phenotype of the sections was observed and photographed using Nikon DS-Fi3 (Nikon) at 100× magnification.

### 2.4 Cell cycle inhibitors examination

Skin tissues were homogenized in a mixture of RIPA protein lysate (Biosharp, BL504A), PMSF (Biosharp, BL507A), and protease inhibitor (Servicebio, G2007-1ML). Total protein in the supernatant was determined using a bicinchoninic acid assay kit (Beyotime Biotechnology Co.), and the corresponding 5 × SDS upwelling buffer (Biosharp, BL502B) was added in a volume ratio of 4:1 (total protein solution: 5 × SDS upwelling buffer). Samples and markers were added, and electrophoresis was performed. In this study, 20 μg of protein was used for analysis. The membrane was then transferred at 250 mA for 60 min. The membrane was rinsed with TBST buffer solution (Servicenio, G0001-2 L) and add QuickBlock™ blocking buffer (Beyotime, P0222). After blocking, the blocking solution was poured off, and 5% skimmed milk powder was prepared in TBST buffer to dilute the p16 antibody (Zenbio, R23896), p21 antibody (Zenbio, 381,102), and p53 antibody (Zenbio, 345,567) at a ratio of 1:1,000, and the GAPDH antibody (Affinity, AF7021) at a ratio of 1:5,000. The antibodies were incubated overnight at 4°C and rinsed with TBST buffer at room temperature on a decolorizing shaker. The secondary antibody (Multi sciences, 70-GAR0072) was diluted in TBST buffer to 1:10,000 and incubated at room temperature for 1 h. After incubation, the membrane was rinsed with TBST buffer at room temperature on a decolorizing shaker. Protein signals were developed using an extra-ultra-sensitive ECL chemiluminescent substrate (Biosharp, BL520B) and visualized with a chemiluminescent imaging system (Clinx, ChemiScope6100). The intensity of Western blot bands was quantified using ImageJ software.

### 2.5 SASP and VRFs analysis

Endothelin-1 (ET-1), interleukin-1β (IL-1β), interleukin-6 (IL-6), interleukin-8 (IL-8), Plasminogen activator inhibitor-1 (PAI-1), and Plasminogen transactivator growth inhibitor (PAI-1β) were detected by using the enzyme-linked immunosorbent assay (Elisa) kits (Elabscience Biotechnology Co., China) at 450 nm.

Nitric Oxide (NO) were detected by using the a colorimetric assay kit (Elabscience Biotechnology Co., China) at 550 nm.

### 2.6 Oxidative stress levels analysis

The total protein concentration of each sample was determined using the enhanced BCA protein assay kit (Beyotime Biotechnology, China), which was used for the calculation of oxidative stress index results.

The inhibition rate of the samples was measured using activity assay kits (Elabscience Biotechnology Co., China) to ensure that the inhibition rate of the samples was between 25% and 45%. The absorbance value of the samples was measured at 412 nm after the enzymatic reaction and color development reaction, and the activity level of glutathione peroxidase (GSH-Px) was calculated.

The OD value of the samples was measured by the WST-1 method at 450 nm using activity assay kits (Elabscience Biotechnology Co., China), and the inhibition rate of Total Superoxide Dismutase (SOD) was calculated. The samples were diluted 100-fold to ensure that the inhibition rate of the samples was between 25% and 65%. The OD values of the samples were measured again, and the SOD inhibition and SOD activity were calculated.

The OD values of the samples were measured by TBA method using a colorimetric assay kit (Elabscience Biotechnology Co., China) at 450 nm, and the levels of malondialdehyde (MDA) were calculated.

### 2.7 Correlation analysis

After obtaining the data of SASP, VRFs, and oxidative stress, correlation analyses were performed using the “Wukong” platform (https://www.omicsolution.com/wkomics/main/).

### 2.8 Statistical analysis

Data entry was performed using Excel 2021 and all statistical analyses were performed using SPSS 26.0. All data information is expressed as *(mean ± SD)*. The significance of differences between groups was determined using the Student’s unpaired t-test, and *P* < 0.05 indicated that the differences were statistically significant.

## 3 Result

### 3.1 HSD has no significant effect on weight in wistar rats

**FIGURE 2:**
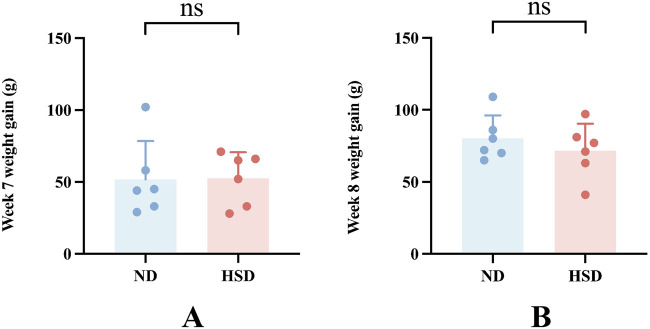
Effect of high salt diet on weight in Wistar rats. **(A)** Weight gain at week 7 compared to the start of the experiment. **(B)** Weight gain at week 8 compared to the start of the experiment.

The weights of the rats were approximately the same before the experiment, and there was no significant difference between the groups (*P*> 0.05). The amount of food given to rats in each group was fixed at 120 g per day, and the weight of rats increased gradually during the HSD. There was no significant difference between the weights of the groups at week 7 and 8 at the time of sampling compared with that at the beginning of the experiment, as shown in Figure 2 and Table 2, indicating that the HSD had no significant effect on the weights of the rats.

**TABLE 2:** Weight at week 7 and week 8 and their gain compared to the start of the experiment.

		ND						HSD					
week 7	weight (g)	218	278	219	239	229	233	235	220	237	248	251	223
	gain (g)	33	58	29	44	45	102	52	28	71	66	65	33
week 8	weight (g)	294	244	245	266	272	250	248	275	263	232	256	245
	gain (g)	109	70	65	72	86	80	71	97	77	41	81	63

### 3.2 HSD mediates differences in aging phenotypes

Previous studies have reported a close relationship between HSD and skin damage. Pajtok found that the HSD was associated with an increased pro-inflammatory state of the dermis and suggested that salt intake may alter the process of skin remodeling (Pajtok et al., 2021). The ECM plays an important role in maintaining the elastic appearance of the dermis and providing a suitable environment for skin cells. With the onset of aging, the ECM undergoes significant changes in morphology and function, mainly in the form of decreased dermal thickness, reduced elasticity, and wrinkle formation (Li et al., 2021). Unfortunately, ECM changes are not a definitive marker of aging. It is currently unclear whether HSD mediates the phenotypic changes in skin aging.

**FIGURE 3:**
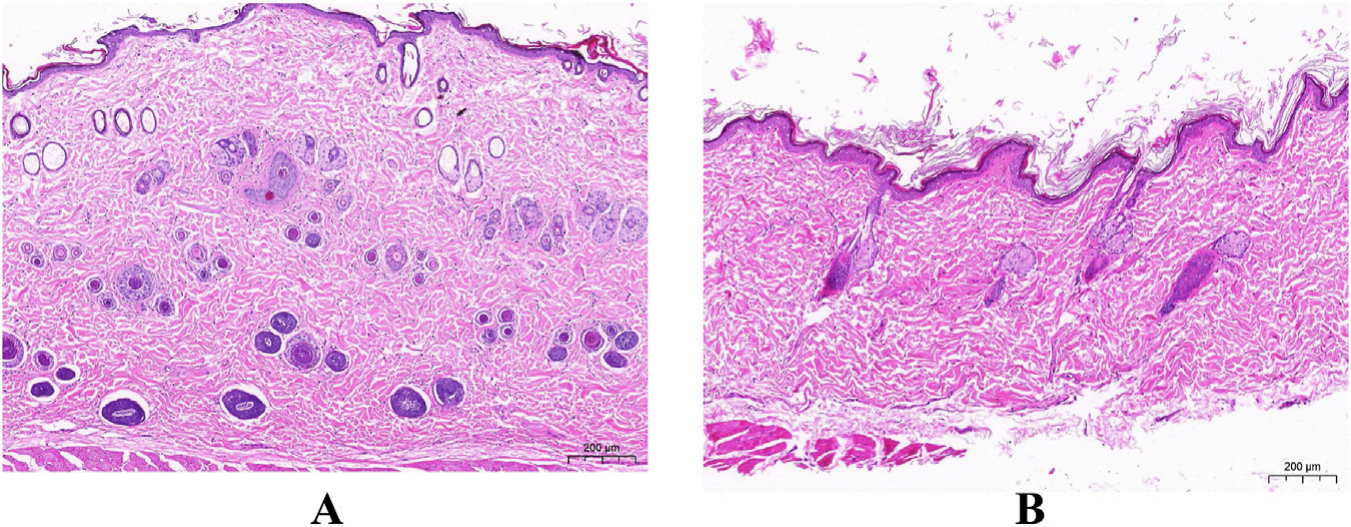
The Pathological section of skin. (HE staining). **(A)** Normal diet group. **(B)** High salt diet group. (Scale bar: A, B = 200 µm).

To clarify this issue, we fed ND (0.3% NaCl) and HSD (8% NaCl) to Wistar rats for 8 weeks. Histological staining of the dorsal skin of rats approximately 4 months old was performed using HE staining to determine the histopathological changes in the skin of rats in the HSD group relative to the skin of rats in the ND group. As shown in Figure 3 and Table 3, HE staining showed that the epidermis of HSD rats fed for 8 weeks was significantly thickened (45.36 ± 14.82 vs. 17.03 ± 5.14) (*P* < 0.01), the dermis was significantly thinned (833.01 ± 138.85 vs. 1341.75 ± 85.27) (*P* < 0.01), and the hair follicles appeared to be atrophied, as compared with ND animals.

**TABLE 3:** Thickness of dermis and epidermis in normal diet and high salt diet groups.

	ND-1	ND-2	ND-3	ND-4	ND-5	ND-6	HSD-1	HSD-2	HSD-3	HSD-4	HSD-5	HSD-6
Epidermal Thickness (um)	15.22	17.91	26.87	14.93	14.93	12.31	38.81	71.89	35.82	53.02	32.00	40.60
Dermal Thickness (um)	1413.76	1191.06	1343.76	1301.83	1406.00	1394.06	705.11	845.67	886.57	941.18	630.54	988.96

### 3.3 HSD induces increased expression of cell cycle inhibitors in the skin

**FIGURE 4:**
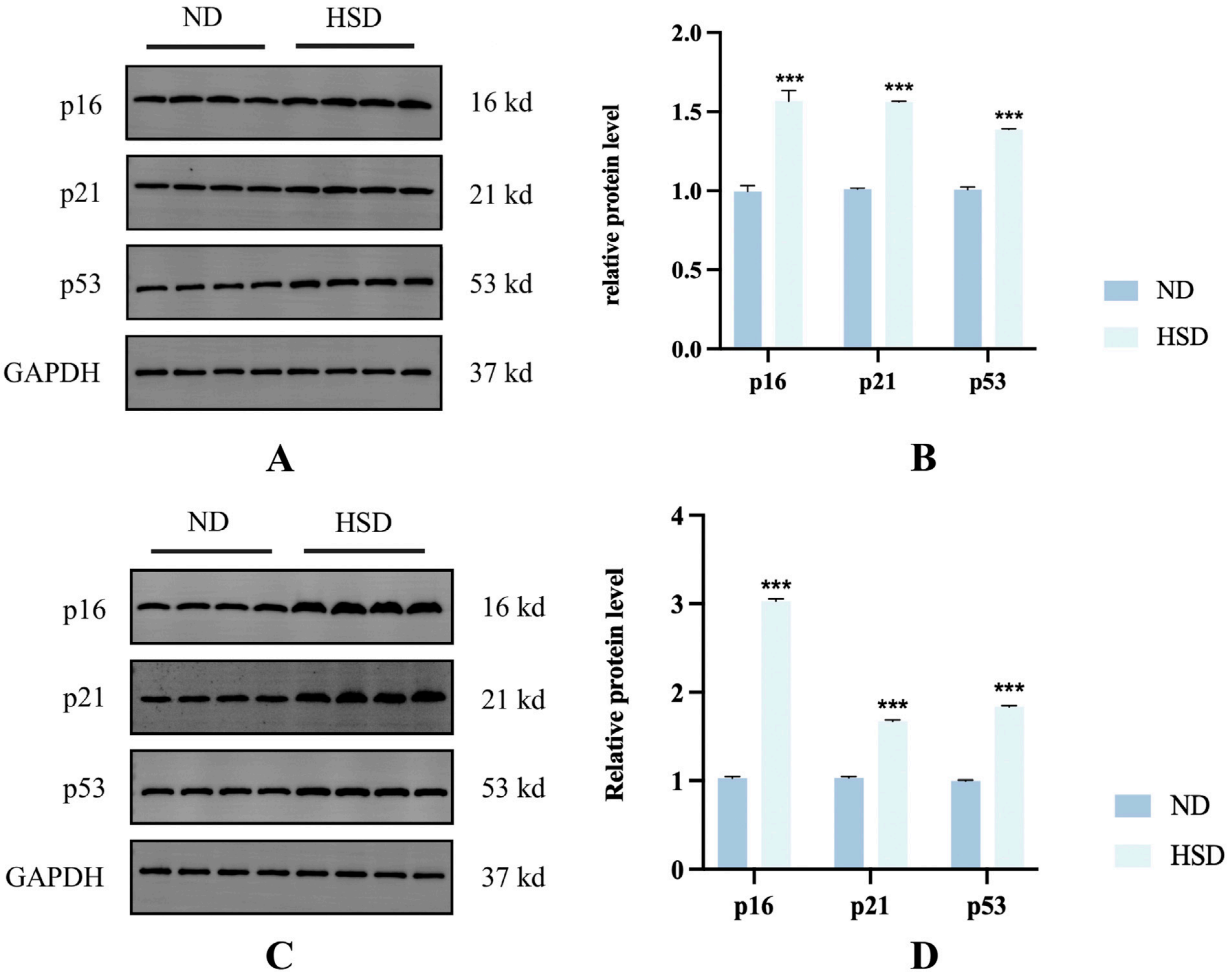
Effect of high salt diet on cell cycle inhibitors in Wistar rats. Expression levels and statistical figures of cell cycle inhibitors in skin tissues induced by normal diet and high salt diet by western blotting (n = 4) **(A, B)** week 7. **(C, D)** Week 8. ***P< 0.001.

The appearance of senescent cells is an important part of aging, and cellular senescence is associated with the aging process in many mammalian tissues, especially the skin (Toutfaire et al., 2017). A key marker of cells entering the senescent state is cell cycle inhibition (Bhatia-Dey et al., 2016). Therefore, cell cycle inhibitors such as p16, p21, and p53 are often used to detect cellular senescence. We detected the cell cycle inhibitory factors p16, p21, and p53 by Western blotting. As shown in Figure 4, HSD induced overexpression of p16, p21, and p53 in the skin at week 7 and week 8 (*P* < 0.001), suggesting that rats in the HSD group showed significant cellular senescence.

### 3.4 HSD induces increased SASP in the skin

SASP has been used as a clinical biomarker of human senescence for the last few years (Hickson et al., 2019; Zhu et al., 2022; Sorokina et al., 2023; Lin et al., 2023). Cellular senescence is a state of stable terminal proliferative arrest, and the detrimental effects of cellular senescence are due to the increased production of SASP (Gorgoulis et al., 2019). The overproduction of SASP is a key feature of cellular senescence and is mediated by multiple pathways. Since cellular senescence has both beneficial and detrimental biological consequences (Wang et al., 2024), identifying SASP in various pathophysiological contexts is important for studying skin aging.

**FIGURE 5:**
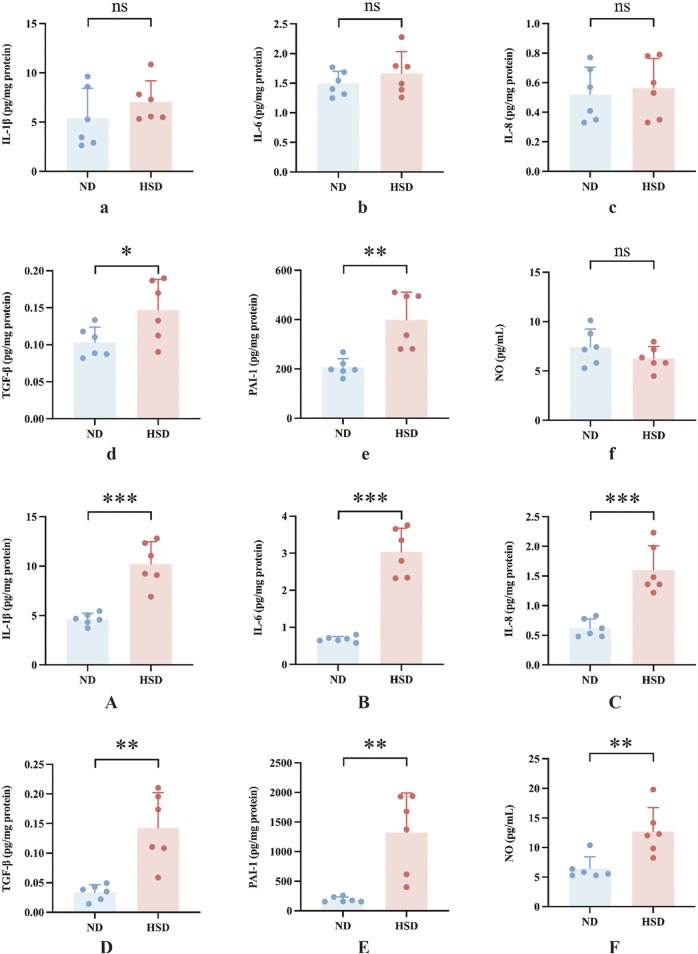
Effects of high salt diet on SASP levels in Wistar rats. SASP is a key marker of skin aging. ELISA was used to measure the levels of **(a)** IL-1β, **(b)** IL-6, **(c)** IL-8, **(d)** TGF-β, **(e)** PAI-1, and **(f)** NO in skin tissue homogenates at week 7, and **(A)** IL-1β, **(B)** IL-6, **(C)** IL-8, **(D)** TGF-β, **(E)** PAI-1, and **(F)** NO at week 8. Values were expressed as *mean ± SD*. Dots represent individual values. (n = 6); *P< 0.05, **P< 0.01, ***P< 0.001 *vs.* normal diet groups (Unpaired t-test).

SASPs are composed of interleukins (e.g., interleukin-1 (IL-1), IL-1β, IL-6), chemokines (e.g., IL-8, TECK), other inflammatory molecules (e.g., TGF-β, GM-CSF), growth factors and regulators (e.g., HGF, EGF), proteases and regulators (e.g., MMPs, PAI-1), non-protein molecules (e.g., PGE2, NO) and a series of cytokines (Gorgoulis et al., 2019). We examined SASP in the skin of rats from the ND and HSD groups at weeks 7 and 8 using ELISA. As shown in Table 4, 5 and Figure 5, at week 7, there were no significant differences in the levels of IL-1β, IL-6, IL-8, and NO between the HSD and ND groups. However, TGF-β (*P* < 0.05) and PAI-1 (*P* < 0.01) levels were significantly increased in the HSD group. By week 8, levels of IL-1β, IL-6, and IL-8 (*P* < 0.001), as well as TGF-β and PAI-1 (*P* < 0.01), were significantly higher in the HSD group compared to the ND group. This indicates that while the HSD did not induce significant inflammatory reactions by week 7, it may have caused some degree of cutaneous fibrosis and hypercoagulation. By week 8, the HSD led to a marked inflammatory response and exacerbated skin fibrosis, contributing to skin aging.

**TABLE 4:** Week 7 SASP data for normal and high salt diet groups and comparison *P v*alues.

	ND	HSD	*P-value*
IL-1β (pg/mg protein)	5.42 ± 3.02	7.06 ± 2.13	0.3024
IL-6 (pg/mg protein)	1.49 ± 0.21	1.66 ± 0.37	0.3451
IL-8 (pg/mg protein)	0.52 ± 0.19	0.56 ± 0.20	0.7052
TGF-β (pg/mg protein)	0.10 ± 0.02	0.15 ± 0.04	0.0429
PAI-1 (pg/mg protein)	206.05 ± 36.18	399.81 ± 111.80	0.0024
NO (pg/mL)	7.43 ± 1.81	6.27 ± 1.21	0.2189

**TABLE 5:** Week 8 SASP data for normal and high salt diet groups and comparison *P v*alues.

	ND	HSD	*P-value*
IL-1β (pg/mg protein)	4.64 ± 0.59	10.23 ± 2.24	0.0001
IL-6 (pg/mg protein)	0.68 ± 0.07	3.04 ± 0.64	0.0001
IL-8 (pg/mg protein)	0.62 ± 0.15	1.60 ± 0.40	0.0002
TGF-β (pg/mg protein)	0.03 ± 0.01	0.14 ± 0.06	0.0013
PAI-1 (pg/mg protein)	189.80 ± 45.11	1322.46 ± 667.88	0.0020
NO (pg/mL)	6.45 ± 1.97	12.72 ± 4.03	0.0065

**FIGURE 6:**
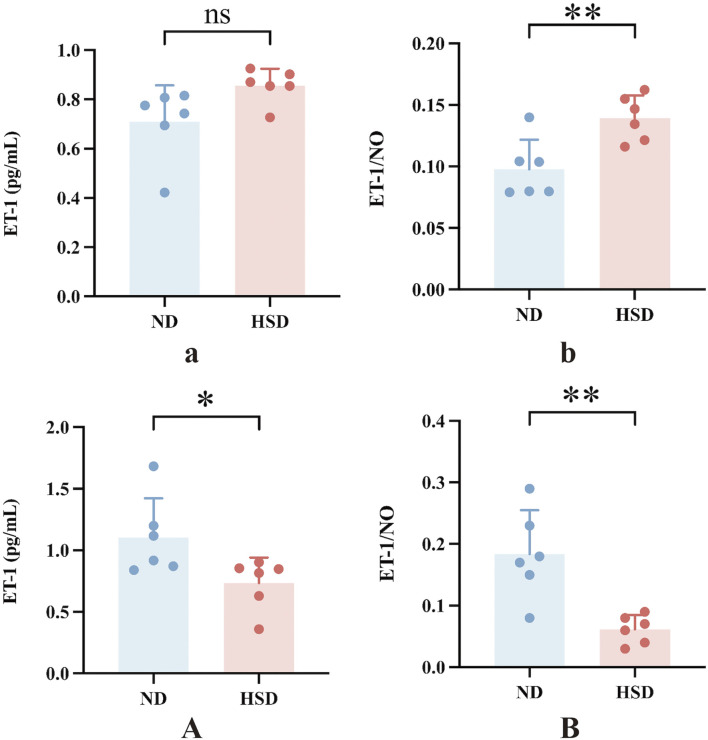
Effect of high salt diet on serum ET-1 and ET-1/NO ratio in Wistar rats. ET-1 can lead to pro-vascular smooth muscle cell division and proliferation and aggravation of ischemia in organs. The alteration of the ratio of ET/NO responds to the contraction of the endothelium of the vasculature and the changes in blood flow in local organs. **(a)** ET-1 levels in week 7, **(b)** ET-1 to NO ratio in week 7, **(A)** ET-1 levels in week 8, **(B)** ET-1 to NO ratio in week 8. Values were expressed as mean ± SD. Dots represent individual values. (*n* = 6); *P < 0.05, **P < 0.01 vs. normal diet groups (Unpaired t-test).

In the present study, PAI-1 and NO levels were significantly elevated in the HSD group at week 8 compared to the ND group. PAI-1 is a mediator and marker of senescence (Wang and Dreesen, 2018), and there is substantial evidence suggesting that PAI-1 plays a decisive role in the fibrinolytic system. While NO acts as a secretory phenotype of aging, it also has a vasodilatory effect and improves blood flow status (Li et al., 2014). To verify whether skin aging affects blood flow status, we further examined ET-1 and calculated the ratio of ET-1 to NO, as shown in Table 6, 7 and Figure 6. At week 7, the ET-1/NO ratio was higher in the HSD group. By week 8, however, serum ET-1 levels were significantly lower in the ND group compared to the HSD group, and the ET-1/NO ratio was also lower in the HSD group. This suggests that HSD may influence blood flow status and vasoconstriction, contributing to skin aging.

**TABLE 6:** Week 7 ET-1 and ET-1/NO data for normal and high salt diet groups and comparison *P v*alues.

	ND	HSD	*P-value*
ET-1 (pg/mL)	0.71 ± 0.15	0.86 ± 0.07	0.0527
ET-1/NO (pg/mL)	0.10 ± 0.02	0.14 ± 0.02	0.0071

**TABLE 7:** Week 8 ET-1 and ET-1/NO data for normal and high salt diet groups and comparison *P v*alues.

	ND	HSD	*P-value*
ET-1 (pg/mL)	1.10 ± 0.32	0.73 ± 0.21	0.0378
ET-1/NO (pg/mL)	0.18 ± 0.07	0.06 ± 0.02	0.0065

### 3.5 HSD induces increased levels of oxidative stress in the skin

ROS are a class of substances composed of or containing oxygen, and under normal conditions, the production and removal of ROS are in a delicate dynamic balance, in which the antioxidant system plays an important role (Obrador et al., 2019). However, when excessive ROS production in the body exceeds the scavenging capacity, it can lead to cellular damage and induction of skin aging.

**FIGURE 7:**
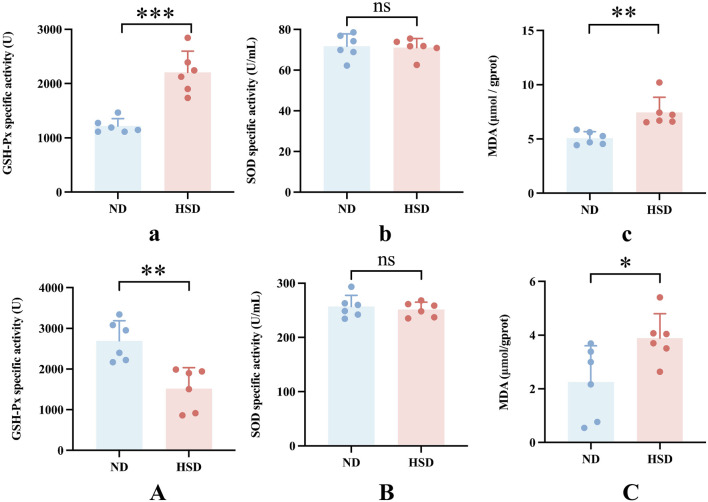
Oxidative stress levels in Wistar rat skin. The rate of aging is linked to ROS production. Oxidative stress was assessed by measuring the specific activity of GSH-Px and SOD, and MDA levels in skin tissue homogenates for week 7 **(a–c)** and week 8 **(A–C)** using colorimetric assays. Values were expressed as *mean ± SD*. Dots represent individual values. (n = 6) in each group; ^ns^ *P* ≥ 0.05 *P < 0.05, **P < 0.01, ***P < 0.001 *vs.* normal diet groups (Unpaired t-test).

We examined the total protein concentration of each sample using the BCA method. We also measured the levels of GSH-Px, SOD, and MDA, which are related to oxidative stress in the skin, using a colorimetric method. GSH, as an endogenous cytoprotectant, plays a major role in the maintenance of intracellular redox state. The activities of GSH-Px and SOD enzymes can reflect the content of intracellular GSH as well as the degree of oxidative damage. MDA is one of the most important products of lipid peroxidation (Kwiecien et al., 2012). The content of MDA in the body can indirectly reflect the degree of free radical damage to the organism and is one of the important indicators for assessing the degree of aging (Gawel et al., 2004). As shown in Table 8, 9 and Figure 7, GSH-Px levels (*P* < 0.001) and MDA levels (*P* < 0.01) were significantly higher in the HSD group at week 7. By week 8, GSH-Px levels had significantly decreased (*P* < 0.01), while MDA levels remained significantly elevated (*P* < 0.05) in the HSD group. There was no significant difference in SOD levels between the two groups. These findings suggest that HSD may initially boost antioxidant capacity, but over time, skin aging results in reduced antioxidant levels. Despite this decrease, free radical damage to the organism continues.

**TABLE 8:** Week 7 Oxidative stress data for normal and high salt diet groups and comparison *p v*alues.

	ND	HSD	*P-value*
GSH-Px specific activity (U)	1217.70 ± 135.72	2207.28 ± 390.79	0.0002
SOD specific activity (U/mL)	71.79 ± 6.02	71.07 ± 4.50	0.8195
MDA (μmol/gprot)	5.07 ± 0.60	7.45 ± 1.39	0.0032

**TABLE 9:** Week 8 Oxidative stress data for normal and high salt diet groups and comparison *p v*alues.

	ND	HSD	*P-value*
GSH-Px specific activity (U)	2693.77 ± 495.09	1518.10 ± 517.19	0.0024
SOD specific activity (U/mL)	256.89 ± 20.81	251.38 ± 13.52	0.5983
MDA (μmol/gprot)	2.25 ± 1.34	3.89 ± 0.91	0.0327

### 3.6 Correlation analysis

**FIGURE 8:**
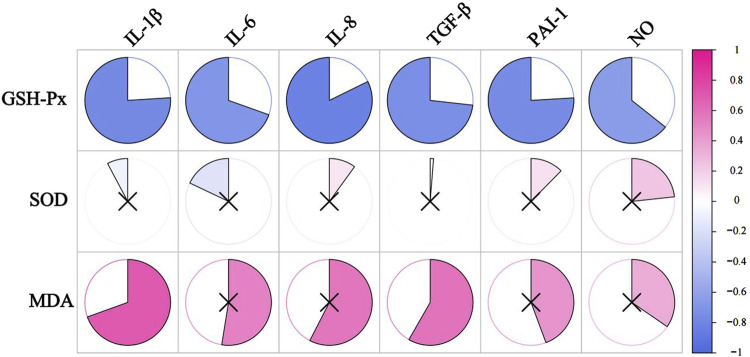
The correlation analysis between SASP and oxidative stress levels. The redder the color, the more positively correlated the two indicators. The bluer the color, the negative correlation between the two indicators. ×represents *P* ≥ 0.05, with no significance.

We correlated SASP with oxidative stress levels in rats that were aged after 8 weeks of HSD feeding, and the *p* values for each metric comparison are shown in Table 10. As shown in Table 11 and Figure 8, there was a significant negative correlation between the level of GSH-Px and the different SASP indexes, indicating that a decrease in GSH-Px may lead to an increase in SASP levels. MDA levels showed a significant positive correlation with IL-1β and TGF-β and a positive trend with IL-6, IL-8, PAI-1, and NO. None of the SOD values correlated with the SASP indexes. The above results suggest a correlation between HSD-induced oxidative stress and the rise in skin SASP indexes due to HSD. Niklander stated that ROS production induces the p38 MAPK pathway, providing stabilization of SASP-encoding mRNA (Niklander et al., 2020). The results of the present study may indicate that high salt leads to a diminished ability of skin tissues to scavenge free radicals, causing an overproduction of ROS and maintaining or enhancing SASP levels.

**TABLE 10:** *P*-value of oxidative stress factors compared to SASP indicators.

	IL-1β	IL-6	IL-8	TGF-β	PAI-1	NO
GSH-Px	0.0041	0.0120	0.0010	0.0068	0.0042	0.0240
SOD	0.8100	0.5764	0.7569	0.9690	0.7014	0.4664
MDA	0.0121	0.0793	0.0503	0.0467	0.1489	0.2714

**TABLE 11:** The data information from the correlation analysis of oxidative stress factors with SASP.

	IL-1β	IL-6	IL-8	TGF-β	PAI-1	NO
GSH-Px	−0.7601	−0.6956	−0.8225	−0.7324	−0.7594	−0.6433
SOD	−0.0779	−0.1796	0.1001	0.0126	0.1238	0.2329
MDA	0.6951	0.5255	0.5754	0.5829	0.4433	0.3454

**FIGURE 9:**
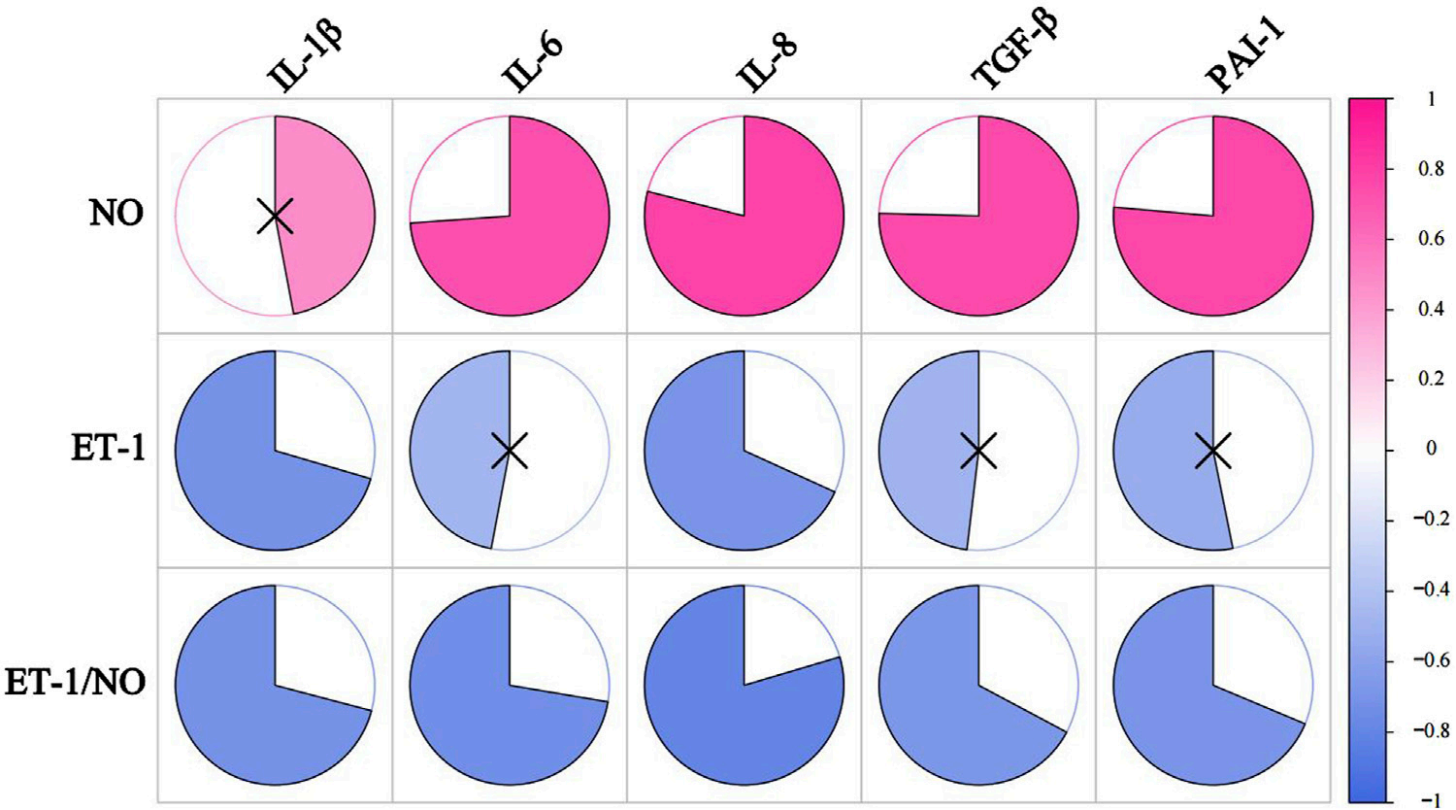
The correlation analysis between VRFs and SASP. The redder the color, the more positively correlated the two indicators. The bluer the color, the negative correlation between the two indicators. ×represents *p* ≥ 0.05, with no significance.

We correlated SASP, other than NO, with vascular regulatory factors (NO, ET-1, ET-1/NO), and the *P*-value between the indicators is shown in Table 12. As shown in Table 13 and Figure 9, the ratio of ET-1/NO had a significant negative correlation with all of the SASP indicators. ET-1 had a significant negative correlation with IL-1β and IL-8, and a tendency to correlate negatively with all other SASP indicators. NO had a tendency to correlate positively with IL-1β, and a significant positive correlation with other SASPs.

**TABLE 12:** *P*-value of VRFs compared to SASP indicators.

	IL-1β	IL-6	IL-8	TGF-β	PAI-1
NO	0.1229	0.0061	0.0022	0.0046	0.0038
ET-1	0.0105	0.1228	0.0146	0.1133	0.0753
ET-1/NO	0.0098	0.0078	0.0020	0.0167	0.0136

**TABLE 13:** The data information from the correlation analysis of VRFs with SASP.

	IL-1β	IL-6	IL-8	TGF-β	PAI-1
NO	0.4703	0.7387	0.7898	0.7544	0.7643
ET-1	−0.7045	−0.4704	−0.6820	−0.4811	−0.5316
ET-1/NO	−0.7090	−0.7235	−0.7959	−0.6721	−0.6868

## 4 Discussion

The skin is divided into three main parts: the epidermis, the dermis, and the subcutaneous tissue. The epidermis is mainly composed of keratinocytes, and the dermis is mainly composed of ECM secreted by fibroblasts, including structural proteins such as collagen and elastin, GAGs, and proteoglycans, which provide mechanical resistance and elasticity to the skin (Arda et al., 2014). The present study showed that in aging skin, the epidermis appeared to be significantly thickened and the dermis significantly thinned. It is suggested that HSD may lead to a decrease in the viability and renewal rate of keratinocytes in the epidermal layer of the skin, a slowing down of collagen and elastin synthesis, and an accelerated breakdown of collagen and elastin in the dermis, resulting in an increase in the thickness of the epidermis and a decrease in the thickness of the dermis.

Cells enter a state of irreversible cell cycle arrest, a process known as “cellular senescence” (Hohn et al., 2017). Stable proliferative arrest is a key feature of senescent cells, and the p53/p21 and p16/pRB pathways are their main regulatory pathways (Coppe et al., 2011; Sturmlechner et al., 2021; Xue et al., 2007). Therefore, the detection of cell cycle regulators p16, p21, and p53 is an important method for detecting cellular senescence. The phosphorylation of p53 has been observed in senescent human cells (Rufini et al., 2013). P21 is regulated by p53 and is the most important factor in the maintenance of cell cycle arrest in G1 cells (Toyoshima and Hunter, 1994). P16 can block cell cycle progression in G1 cells and prevent cells from moving from the G1 phase into the S phase, where most senescent cells remain (Nevins, 2001). The appearance of senescent cells is an important part of the skin aging process, and previous studies have reported a close relationship between cellular senescence and skin aging (Velarde et al., 2012). Cellular senescence is involved in age-related diseases of the skin. Quan (Quan et al., 2012) found that senescent cells lead to collagen abnormalities, and Gannon (Gannon et al., 2011) found that senescent cells also lead to a progressive loss of epidermal stem cell function. In the present study, there was a significant increase in the expression of p16, p21, and p53 at week 7 and week 8, suggesting the accumulation of senescent cells in the skin after HSD.

Senescent cells are characterized by their inability to proliferate, their resistance to apoptosis, and their ability to secrete factors that promote inflammation and tissue deterioration. This particular secretory state, known as the SASP, mediates many of the cell’s physiological and pathological effects (Cuollo et al., 2020). Because senescent cells have both beneficial and detrimental biological consequences, the detection of SASP is important to investigate whether HSD contributes to skin senescence. SASP includes interleukins, chemokines, other inflammatory molecules, and non-protein molecules (e.g., IL-1β, IL-6, IL-8, TGFβ, PAI-1, NO). Studies have shown that these cytokines lead to collagen breakdown and loss of elasticity (Lee et al., 2021). The results of this study showed that SASP indicators, including inflammatory factors, chemokines, and growth factors, were significantly elevated in skin tissues after 8 weeks of HSD.

The inflammatory response is an important mechanism associated with aging, and individuals continue to experience different categories of pro-inflammatory states with age (Ferrucci and Fabbri, 2018). This chronic, low-grade inflammatory response with age has been termed “inflammaging” (Gibon et al., 2017). By week 8, the levels of IL-1β and IL-6 were significantly higher in the HSD group compared to the ND group in this study, indicating that skin aging associated with high salt intake is closely related to inflammation. IL-1 can activate NF-κB through binding to IL-1 receptor/Toll-like receptor, further increasing SASP expression (Chang et al., 2002) and exacerbating skin aging. IL-6 has been associated with DNA damage-induced senescence in fibroblasts, melanocytes, and keratinocytes (Lu et al., 2006). Fibroblast-derived IL-6 is responsible for dermal mesenchymal collagen catabolism (Wlaschek et al., 1994), and IL-1β plays an important role in the production of IL-6. This indicates that HSD induces skin SASP production and an inflammatory response by week 8. The elevated levels of IL-1β and IL-6 contribute to skin cellular senescence and the breakdown of the extracellular matrix. Additionally, the increase in inflammatory factors such as IL-1β and IL-6 due to HSD further enhances SASP expression.

TGF-β also plays an important role in the control of anti-inflammatory and pro-inflammatory T-cell responses, and TGF-β is an important regulator of tissue fibrosis, as well as a marker of tissue senescence (Ren et al., 2023; Chen D. Q. et al., 2018; Chen L. et al., 2018). Castello stated that in fibrosis related to aging, there is a senescent cell long-term abnormal accumulation and a long-term increase in low-grade inflammation (Castello et al., 2010). The results of this study revealed that TGF-β levels were elevated in the HSD group at week 7 and increased further by week 8. By week 8, during the occurrence of skin senescence, the levels of inflammatory cytokines, including IL-1β, IL-6, and TGF-β, were significantly higher in the HSD group compared to the ND group. These inflammatory cytokines can recruit immune cells such as mast cells, B cells, T cells, and macrophages, and the immune cells can release IL-1β, IL-6, and TGF-β again to enhance fibrosis and inflammatory response (Erazo-Martínez et al., 2023). It suggests that during the process of skin aging due to HSD, a continuous aggravation of inflammatory response and fibrotic changes may occur, thus further accelerating skin aging.

PAI-1 is a major target gene of TGF-β1/p53 and plays a role in triggering aging downstream of p53 (Kortlever et al., 2006). In this study, both TGF-β and PAI-1 were significantly elevated in the HSD group at weeks 7 and 8, suggesting that HSD-induced skin aging may be linked to the TGF-β1/P53/PAI-1 signaling axis. And in the process of HSD-induced skin aging, decreased activity of the fibrinolytic system and hypercoagulation may occur.

NO, as a component of SASP is also a vasodilator substance, which prevents thrombosis (Förstermann and Sessa, 2012). NO also has an antiproliferative effect on vascular wall cell proliferation, reduces the permeability of the large blood vessels and the resistance vessels, and improves the blood flow condition (Li et al., 2014). The results of the present study showed a significant increase in NO levels in the HSD group, suggesting that pro-blood flow increases may occur in conjunction with skin aging. This may seem to contradict the elevated PAI-1 levels in the HSD group. To investigate how HSD alters skin blood flow status, we examined ET-1 levels in our samples. Endothelin (ET) has four isomers, ET-1, ET-2, ET-3, and ET-4, of which ET-1 is the most biologically active, with strong vasoconstrictive, positive inotropic effects, leading to pro-vascular smooth muscle cell division and proliferation and aggravation of ischemia in organs (Davenport et al., 2016). The alteration of the ratio of ET/NO responds to the contraction of the endothelium of the vasculature and the changes in blood flow in local organs. In this study, at week 7, the ET/NO ratio was elevated in the HSD group. By week 8, the ET/NO ratio was significantly lower in the HSD group compared to the ND group. This change may be attributed to vascular endothelial damage caused by the HSD in the first 7 weeks, leading to decreased NO levels and increased ET-1 levels. However, by week 8, the skin aging occurred, and NO, one of the SASP indicators, increased significantly in the HSD group.

It has been shown that the rate of aging is related to the production of ROS (Richter and Kietzmann, 2016). When the body produces more ROS than it can remove, ROS can directly damage lipids, proteins, *etc.*, and further contribute to skin aging (Kammeyer and Luiten, 2015). In addition, ROS are capable of degrading components such as elastin and collagen in the ECM (Pittayapruek et al., 2016). MDA, SOD, and GSH-Px are recognized as key markers of oxidative stress (Abedi et al., 2023). Under normal conditions, the antioxidant system plays an important role in the dynamic balance of ROS production and removal (Obrador et al., 2019). The most important antioxidant enzyme for the elimination of free radicals is GSH-Px, an antioxidant enzyme that removes monoclinic oxygen, H_2_O_2_, and organic peroxides by reacting with the sulfhydryl groups of glutathione (Zachara, 2015). SOD catalyzes the disproportionation reaction of superoxide to O_2_ and H_2_O_2_, indirectly reflecting the body’s ability to scavenge oxidative free radicals (Mo et al., 2022). MDA directly damages biological macromolecules and cellular membranes, and its content MDA can directly damage biomolecules and cell membranes, and its content is closely related to lipid peroxidation in the organism (Ma et al., 2019). Studies have shown that the level of MDA is positively correlated with increasing age, and is one of the most important indicators for assessing the degree of aging (Gawel et al., 2004). The results of the present study demonstrated that GSH-Px levels were significantly increased at week 7 but significantly decreased by week 8. MDA levels were significantly elevated at both week 7 and week 8. This suggests that HSD may initially trigger a stress response, leading to an increase in antioxidant capacity. However, as skin aging progresses, antioxidant capacity declines. Despite the initial response, free radical damage persisted, leading to an imbalance in ROS accumulation and scavenging, which ultimately contributed to skin aging.

In the analysis of the correlation between SASP indicators (excluding NO) and vascular endothelial damage factors measured at week 8, the results indicated that PAI-1, a key inhibitor of fibrinolytic activity, showed a positive correlation trend with NO, a negative correlation trend with ET-1, and a significant negative correlation with the ET-1/NO ratio. There are two possible reasons for this result. Firstly, NO, a key indicator of the SASP, increases in skin aging. However, its bioefficacy may decline due to reduced antioxidant capacity and elevated oxidative stress. Secondly, although aging skin has fewer blood vessels, the existing capillaries may experience increased blood flow. Li (Asahi et al., 1995) stated that capillary collaterals are the main source of nutrients to the skin as the superficial dermal layer decreases with atrophy. Consistent with this study, they found that capillary collaterals in the dermal papillae decreased with age, while capillary spacing and blood flow increased (Li et al., 2006). It has also been shown that the vascular system of aging skin is relatively devoid of blood vessels, with a 35% reduction in venous cross-sectional area in older skin compared to younger skin (Gilchrest et al., 1982). Therefore, in this study, HSD resulted in a simultaneous increase in blood flow and a tendency for blood to become hypercoagulable in aging skin, possibly due to dermal atrophy and a reduction in vascularity during skin aging. In order to provide nutrients to the aging skin, some protective mechanism exists to induce vasodilation and increased blood flow.

In the correlation analysis of SASP and oxidative stress indicators measured at week 8, we found that SASP levels were significantly negatively correlated with GSH-Px and positively correlated with MDA. This indicates that cellular senescence caused by HSD is negatively correlated with antioxidant capacity and positively correlated with damage caused by free radicals. A variety of inflammation-associated molecules are present in SASP, and Sies indicated that ROS production can also trigger an inflammatory response through the activation of NF-κB (Sies et al., 2017). Senescence-induced ROS have been found to promote the transcription of the senescence-associated IL-6 gene (Zhang et al., 2004) and induce elevated levels of IL-8 in mice (Dong et al., 2017). Sheng (Sheng et al., 2013) demonstrated that IL-1β activation can increase the level of 8-isoprostane, an indicator of ROS damage. Additionally, ROS are essential for the release of mature IL-1β. This suggests a bidirectional relationship, while IL-1β activation leads to increased ROS damage, ROS are also required for IL-1β maturation. Richter found that oxidative stress activates TGF-β signaling, and TGF-β1 inhibits the expression of several antioxidant enzymes, thereby increasing ROS formation (Richter and Kietzmann, 2016). Based on this, the present study shows that oxidative stress during skin aging interrelates with cellular senescence-induced inflammatory responses. Excessive ROS produced by HSD induces elevated expression of IL-6 and IL-8 and plays an important role in the release of mature IL-1β. Meanwhile, high levels of IL-1β and TGF-β may lead to a decrease in the antioxidant capacity of the skin, aggravate free radical-induced damage, and further aggravate oxidative stress in the skin. In this study, PAI-1 levels were positively correlated with oxidative stress levels. Jiang found that H_2_O_2_ stimulates PAI-1 expression and inhibits fibrinolytic activity by depleting intracellular GSH (Jiang et al., 2003). It suggests that HSD may exacerbate oxidative stress, thereby inducing skin cellular senescence and weakened anticoagulant function. In this study, NO levels in rat skin at week 8 were positively correlated with oxidative stress. Aging characteristics include the activation of inducible nitric oxide synthase (iNOS) and endothelial nitric oxide synthase (eNOS), impaired antioxidant activity, and increased oxidative stress. The latter two factors reduce NO bioavailability and lead to endothelial dysfunction (Zanetti et al., 2010). NO has a dual effect on GSH-Px. On one hand, it can induce the expression of GSH-Px family members (Castella et al., 2017). On the other hand, it can inactivate GSH-Px by directly binding to its amino acid residues, leading to increased peroxide accumulation (Asahi et al., 1995). Our findings show that elevated NO levels are negatively correlated with GSH-Px levels and positively correlated with MDA levels. This suggests that in aging rats, HSD increases NO levels, which inhibits GSH-Px, exacerbates peroxide accumulation, and raises MDA levels. However, the overall bioefficacy of NO may decrease during skin aging due to diminished antioxidant capacity and heightened oxidative stress.

## 5 Conclusion

In conclusion, HSD leads to skin aging and elevated levels of oxidative stress. Our results demonstrate that continuous intake of a HSD causes an accumulation of senescent cells in the skin and induces overexpression of cell cycle inhibitors p16, p21, and p53. After 8 consecutive weeks of high salt intake, there is a notable elevation in the SASP, resulting in significant skin aging. This study provides theoretical support for optimizing the timing of salt-restricted dietary interventions to delay skin aging.

## Data Availability

The original contributions presented in the study are included in the article/supplementary material, further inquiries can be directed to the corresponding author.
